# Supplementary material for: Fluorescence changes in carbon nanotube sensors correlate with THz absorption of hydration
Source: Nat Commun. 2024 Aug 8;15:6770. doi: 10.1038/s41467-024-50968-9 (PMC11310214; doi:10.1038/s41467-024-50968-9)
Supplement: Supplementary file 1 — Supplementary Information [file 41467_2024_50968_MOESM1_ESM.pdf]

Supporting Information for

THz coupling in carbon nanotube sensors modulates their fluorescence

Sanjana S. Nalige[1], Phillip Galonska[1], Payam Kelich[2], Linda Sistemich[1], Christian Herrmann[3], Lela Vukovic[2], Sebastian Kruss[1,4*], Martina Havenith[1*]

[1]Department of Physical Chemistry II, Ruhr University Bochum, 44801 Bochum, Germany

[2]Department of Chemistry and Biochemistry, University of Texas at El Paso, TX 79968, USA

[3]Department of Physical Chemistry I, Ruhr University Bochum, 44801 Bochum, Germany

[4]Fraunhofer Institute for Microelectronic Circuits and Systems, 47057 Duisburg, Germany.

*Corresponding authors: Sebastian Kruss, Martina Havenith.

Email: sebastian.kruss@rub.de, martina.havenith@rub.de

This PDF file includes:

Supporting text
Figures S1 to S16
Tables S1 to S3
SI References

Supporting Information Text

1. NIR Spectroscopy Data

The NIR spectroscopy data are shown in Supplementary Fig. 2, 3, 5, and 6.

2. Isothermal Titration Calorimetry (ITC)

We used Isothermal Titration Calorimetry (ITC) to access further information about the sensing characteristics of dopamine by SWCNTs. For this purpose, dopamine was titrated to $(GT)_{10}$-SWCNTs up to a final concentration of around 180 µM. Various SWCNT concentrations (25 - 200 nM) were tested, resulting in a similar profile of the thermogram. Small amounts of dopamine, i.e., the first injections result in a pronounced exothermic response, followed by smaller heat changes for subsequent injections (Supplementary Fig. 9). As a control experiment, dopamine was titrated to free single-stranded $(GT)_{10}$-DNA in the cell resulting in small and constant heat changes due to the dilution of dopamine (panel f of Supplementary Fig. 9). Another control experiment with buffer only in the cell showed the same small and constant heat changes (Supplementary Fig. 9-g).

The ITC experiments suggest that dopamine finds strong and weak binding sites on the $(GT)_{10}$-coated SWCNTs. The large changes, in the beginning, originate from a strong binding site that is saturated by dopamine after a few injections. The weak binding site yields small and constant heat changes of -0.2 kcal/mol until the end of the titration meaning that saturation cannot be reached due to the weak interaction. To evaluate this binding isotherm with a one-site binding model this second, weak binding effect (-0.2 kcal/mol) was subtracted from the data. Thus, the lower panel of (a) – (e) in Supplementary Fig. 9 shows the plot of the heat released (after subtraction of the weak effect) against the ratio of the concentrations of dopamine and SWCNTs together with the one-site fit. Most intriguingly, this fit yields insight into the stoichiometry of dopamine with the SWCNTs. We deduce an interaction stoichiometry ranging from 1:30 to 1:43 (SWCNTs:dopamine, Supplementary table 3), meaning that an SWCNT covered with ssDNA harbors on average 37 strong interaction sites for dopamine. This value is different from the published number of ssDNA molecules on a SWCNT, being around 10x higher[1] .This discrepancy can be attributed to the fact, that the ssDNA is not uniformly distributed on the SWCNT's surface, and therefore not each single DNA molecule adopts an adequate conformation to allow interaction with dopamine. Treating the binding isotherms as a one-site binding model, the dissociation constant of the tight dopamine binding site was determined to be 11 µM (Supplementary Table 3).

This thermodynamic dissociation constant (K_D) differs from already published values obtained from fluorescence titrations[2]. On the one hand, these properties are extremely sensitive to changes in the SWCNT's corona, which can already be induced by weak/small interactions of the analyte with the SWCNT. On the other hand, it needs to be considered, that the change in fluorescence is not proportional to the density of analytes interacting with the nanotube. Therefore, the thermodynamic K_D is higher, than the apparent K_D obtained from fluorescence measurements.

The smaller ITC peaks indicate an additional weak interaction of dopamine with the $(GT)_{10}$ -functionalized SWCNTs. Control measurements of dopamine titration in PBS (Supplementary Fig. 9-g) and dopamine titration in free ssDNA $(GT)_{10}$ in the absence of SWCNTs (Supplementary Fig. 9-f) resulted in similar thermograms and therefore clearly show, that no interaction of dopamine with free ssDNA is detectable. The observed peaks in the titration of dopamine to DNA-coated SWCNTs can neither be accounted to the dilution of dopamine nor to a possible interaction of dopamine with free DNA. This weak interaction cannot be characterized in quantitative terms because the changes are small, and they do not reach saturation.

Titration experiments were performed using a MicroCal VP-ITC (Malvern Instruments, Malvern UK). The syringe was loaded with a 1 mM solution of dopamine in 1x PBS pH 7.4 and the sample cell was loaded with 200 µl of a 25-29 199 nM solution of $(GT)_{10}$-SWCNTs or 35 µM of free single-stranded $(GT)_{10}$-DNA in 1x PBS pH 7.4. The titration experiments were performed at 25 °C with 23-27 injections of 10 µl into the stirred cell. Data were analysed using Origin software (Microcal Inc.). By fitting a single-site binding model the dissociation constant K_D was determined[3].

3. Molecular dynamics (MD) simulations

Initial atomistic models of ssDNA-SWCNT conjugates

A segment of a (6,5) single-walled carbon nanotube, 34 nm in length, was built with the Carbon Nanostructure Builder plugin in VMD software. One molecule of $(GT)_{10}$ ssDNA polymer was built in a helical conformation around the (6,5) SWCNT using Material Studio, as previously described in Ref [4] with ssDNA bases not being pre-adsorbed to the SWCNT surface. Then, a total of eight $(GT)_{10}$ molecules in pre-built helical conformations were placed along the (6,5) SWCNT, resulting in the initial structures shown in Supplementary Fig. 8a.

Models of ssDNA-SWCNT conjugates with a higher density of ssDNA

Based on previous experimental measurements of DNA density on SWCNT surfaces[1] , each adsorbed $(GT)_{10}$ molecule spans ~ 1.5 nm of (6,5) SWCNTs length on average. Based on these measurements, eight $(GT)_{10}$ molecules should adsorb on 12 nm long (6,5) SWCNT. To increase the density of $(GT)_{10}$ DNA, we solvated the above-prepared system in TIP3P water and simulated it with forces applied on DNA molecules, implemented via the collective variable calculations in NAMD2.13 software[5]. Harmonic forces were progressively applied to the backbone of DNA molecules, causing them to move from their initial positions toward the target positions necessary for absorption onto a 12 nm long (6,5) SWCNT (Supplementary Fig. 8b). The entire simulation process took 175 nanoseconds to reach the final structure. The initial force constant was set at 1 kcal/mol/Å^2 and was increased by 0.5 kcal/mol/Å^2 every 50 nanoseconds. However, during the final 25 nanoseconds, the force constant was adjusted to 3 kcal/mol/Å^2 (Supplementary Fig. 8c). The simulations were carried out using Langevin dynamics, employing a Langevin constant of γ_{Lang}= 1.0 ps^{-1}. A constant temperature and pressure were maintained at 310 K and 1 bar, respectively, with pressure adjustments allowed along the z-axis. Long-range Coulomb interactions were computed using the particle mesh Ewald (PME) method[6] , with periodic boundary conditions used in all dimensions. A time step of 2.0 fs was used. Van der Waals and explicit Coulomb interactions were performed everyone- and two-time steps, respectively.

Once the ssDNA was packed along the 12 nm long segment of the initial SWCNT, the 34 nm long initial SWCNT was replaced with a 12 nm long final SWCNT. This final system, with the final density of one $(GT)_{10}$ molecule per 1.5 nm length of (6,5) SWCNT, was used as a starting point for further simulations. This system as prepared formed a control system, containing eight $(GT)_{10}$ molecules adsorbed on 12 nm long (6,5) SWCNT that was solvated in TIP3P water and 0.155 M NaCl, prepared using the solvate and ionize VMD plugins[7].

Models of ssDNA-SWCNT conjugates with the added dopamine and riboflavin ligands

Two additional systems were prepared and were each investigated in two independent simulations. Each system contained either of the two types of ligands, dopamine (DA) and riboflavin (RBF), where ten of each ligand molecule were distributed randomly around the control DNA-SWCNT conjugate in the aqueous solution. All the added ligands were within ~ 1 nm of the DNA corona. After the distribution of ligands, the prepared systems were solvated in TIP3P water and 0.155 M NaCl, prepared using the solvate, and ionize VMD plugins. The numbers of atoms and system sizes are summarized in Supplementary Table 2.

MD simulation details

To simulate the systems, we employed atomistic MD simulations and used the CHARMM36 parameters[8] . Dopamine and riboflavin parameters were based on the CHARMM general force field[9] . The NAMD2.13 package was utilized to run the production run simulations, using the same parameters as described above in section 1.2. The control system (1 independent run) and two systems with dopamine and riboflavin (two independent runs each) were equilibrated for 3 μs, with only the SWCNT atoms being restrained. In each ligand system, the collective variable (colvar) approach was utilized for one system to prevent ligands from binding to DNA-SWCNT system near the edges of SWCNTs. An additional restraint potential, V, was applied on the x-coordinate of all atoms of the ligands, x_{ALL} of each ligand:

$$V = \begin{cases} \frac{1}{2}k\left(x_{ALL} + 50\ \text{Å}\right)^2, & x < -50\ \text{Å} \\ 0, & -50\ \text{Å} < x < 50\ \text{Å} \\ \frac{1}{2}k\left(x_{ALL} - 50\ \text{Å}\right)^2, & x > 50\ \text{Å} \end{cases} \quad (1)$$

where the value of the spring constant k was set to 3 kcal/(mol·Å^2).

Computational Data Analyses

Two analyses were performed for bulk systems and for local selections of atoms in each system. These analyses include the contact area between SWCNT and selected moieties, and the mass center distance between dopamine and SWCNT.

Mass center distance analysis

To characterize the binding nature of ligands to DNA-SWCNT conjugates, we computed the distances of all the ligands from the SWCNT mass center in the yz plane. The distance of a ligand molecule i from the SWCNT mass center was determined using the following equation:

$$d_i(t) = r_i(t) - r_{SWNT} \quad (2)$$

where $r_i(t)$ is the Euclidean distance of the center of mass of a ligand i at time t, defined in the cylindrical coordinate system, centered around the SWCNT, whose cross section is defined in the yz plane.

Analysis of contact area between SWCNT and water

The following equation determines the contact area between the selected moiety A and the SWCNT surface:

$$\mathrm{S}_{\mathrm{contact-area}}(\mathrm{t}) = \frac{(\mathrm{S}_{\mathrm{SWCNT}}(\mathrm{t}) + \mathrm{S}_{\mathrm{A}}(\mathrm{t})) - \mathrm{S}_{\mathrm{SWCNT\ or\ A}}(\mathrm{t})}{2} \quad (3)$$

where, $\mathrm{S}_{\mathrm{SWCNT}}(\mathrm{t}), \mathrm{S}_{\mathrm{A}}(\mathrm{t})$ and $\mathrm{S}_{\mathrm{SWCNT\ or\ A}}(\mathrm{t})$ represent solvent accessible surface areas (SASA) of SWCNT, A molecules and SASA of both SWCNT and A molecules at time t, respectively. In our calculations, the selected moieties of interest were water, ions, and ligand molecules. The results of the contact area analysis are shown in Supplementary Fig. 11.

Contact areas were obtained both for the whole system and for selected local regions. For example, contact areas were calculated between the whole SWCNT and either water, ions or bound ligands. Separately, contact area analyses were also performed in local regions of SWCNT surface where ligands were observed to bind for at least 400 ns. The local regions were defined as atoms of the SWCNT surface located within a defined distance from the bound ligand, the distance being 8 Å plus the average distance of each atom from the mass center of the ligand were selected. (2) was then used to evaluate the contact area between the local SWCNT region and water.

The contact area between the ligand and the local region was calculated throughout the entire trajectory to identify the instances when the ligand covered the local region. The resulting contact

area values were sorted, and the median was determined to serve as a threshold. Contact areas below the threshold were classified as non-binding, while those above the threshold were considered binding events. Finally, bar plots were created to display the water contact area for non-binding and binding times, shown in Supplementary Fig. 12. The height of each bar was represented by the average contact area, while the standard deviation was indicated by the error bars. All the bash script and Python codes used for analyses are available on GitHub website (https://zenodo.org/records/12667707).

4. Fourier Transform IR - THz Spectroscopy

Instrumentation

FTIR-THz measurements were conducted with a mercury vapor lamp as the source, a mylar multilayer beam splitter, and a liquid helium-cooled silicon bolometer (Infrared Laboratories, Tucson, AZ) detector (Supplementary Fig. 13). The spectrometer compartment was kept under vacuum (3 mbar), while the sample compartment was kept under N_2 purged (1.5 bar) condition during measurements. The sample compartment was separated by polyethylene flaps from the rest of the evacuated spectrometer chamber. The precise layer thickness was determined by recording the etalon fringes of the empty cell by mid-infrared absorption spectroscopy. Each spectrum was measured as an average of 64 scans with a frequency resolution of 2 cm^{-1}.

The total absorption co-efficient (α) and difference absorption spectra ($\Delta\alpha$) is calculated as below:

$$\alpha = -\frac{1}{d} \log\left(\frac{I}{Io}\right) \qquad (4)$$

'd' is the measured layer thickness, I and I_0 being the transmitted intensities of the sample and background, respectively. Before and after each measurement, we carried out a reference measurement with an empty cell. The spectra in the main manuscript are the difference spectra Δα before and after the addition of either the DNA-SWCNT (referenced to water) or upon the addition of analyte (referenced to water). These were corrected for background (empty cell) and water vapor.

THz spectra of DNA-SWCNT with and without analytes

The DNA-SWCNT samples were measured before and after the addition of analytes (waited 15 to 30 minutes to equilibrate after addition). All spectra in the manuscript show the difference spectra Δα of before and after the addition of analytes referenced to water. These were corrected for background and water vapor. Two DNA sequences namely $(GT)_{10}$ and $(AT)_{15}$ were used. Each of these sequences wrap in a slightly different way around the SWCNTs and the fluorescence response varies based on the DNA sequences. Hence, we expected to also see changes in our THz spectra as the coupling between charge carriers of SWCNTs and water is affected by changes in the local solvation shell. This coupling in the case of each DNA sequence should further change with the addition of the analytes. We do observe the same in our FTIR - THz experiments (Supplementary Fig. 1 and Supplementary Fig. 4). But the direction of change in THz intensity (Δα) remains the same for each analyte irrespective of the DNA sequence i.e., for dopamine, it always decreases and for riboflavin, it always increases.

THz spectra of buffer with and without analytes

Supplementary Fig. 14 displays the control measurements of just the PBS buffer and PBS buffer + analytes. We did not see a broadband increase/decrease in the THz intensity on the addition of analytes which corroborates our theory that broadband changes in intensity in our THz spectra are from the coupling between various charge carriers in the SWCNTs sample with local water molecules around them.

Time-dependent measurements of analytes in the buffer

Analytes tend to polymerize or become unstable (in varied conditions based on the analyte) overtime. To exclude a possible influence of overtime changes in the analytes-PBS buffer solution to the change to the THz intensity we performed time-dependent FTIR-THz measurements of the analyte-buffer solution (Supplementary Fig. 15). The same sample cell and analysis procedure was used. The buffer solution was added to the analyte and this solution was then injected into the sample cell in less than two minutes and measured over time. We saw that each analyte took somewhere between 2 to 30 minutes to equilibrate and after that, we did not observe any changes associated with the analyte or buffer solution itself.

THz spectra of solid SWCNTs and dried DNA-SWCNT

Supplementary Fig. 16 is the plot of dry SWCNT measured with an ATR (attenuated total reflection) FTIR unit (because the sample is solid and cannot directly be measured in the standard transmission cell with two diamond windows)

Figures and Tables

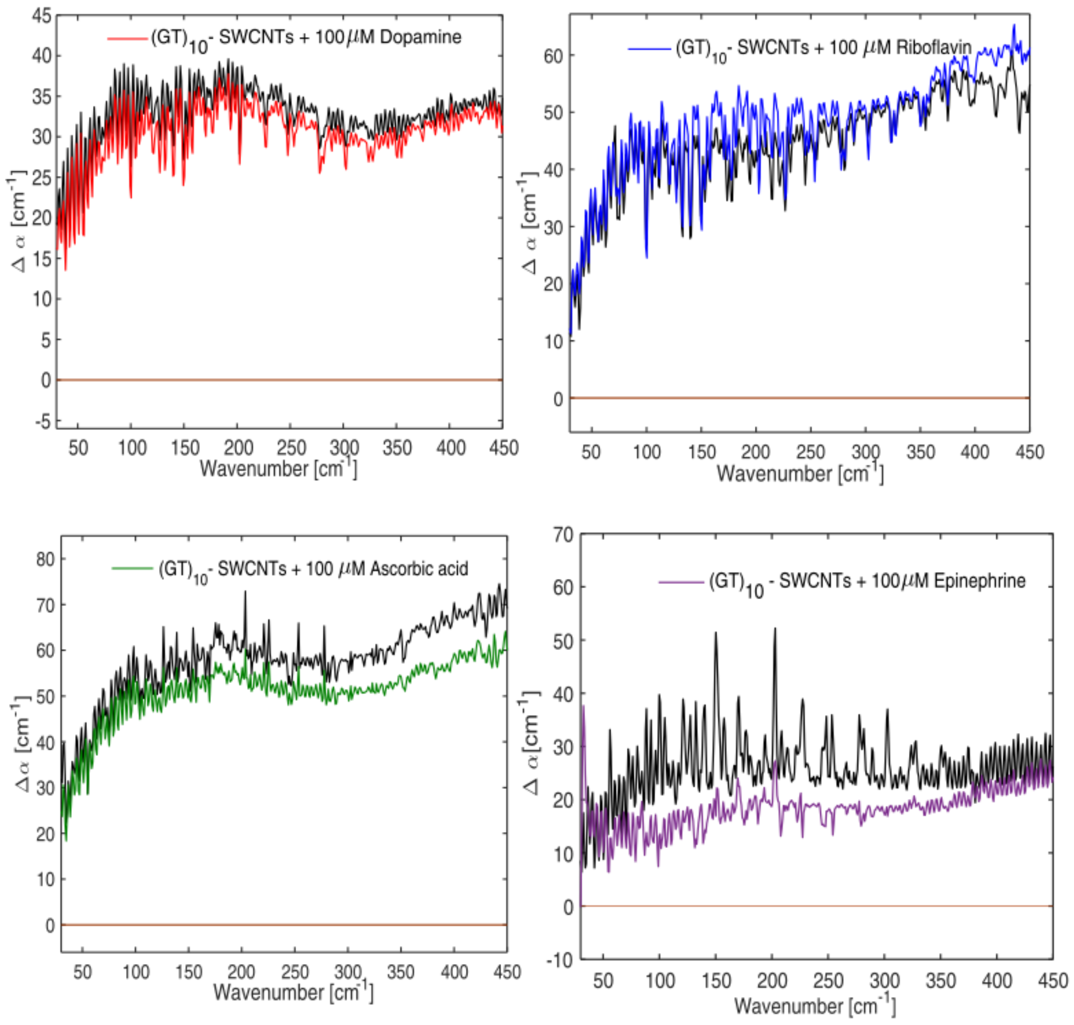

Supplementary Fig. 1. Plot of difference spectra, $\Delta\boldsymbol{\alpha}$ (= $\boldsymbol{\alpha}_{sample} - \boldsymbol{\alpha}_{water}$) vs wavenumber of DNA-SWCNTs(black) and of them with analytes (color coded). The DNA sequence used here is $(GT)_{10}$. The above spectra are not corrected for concentration. They are an average of multiple trials of distinct samples carried out on two days for each analyte. Concentration corrected data set available at the repository.

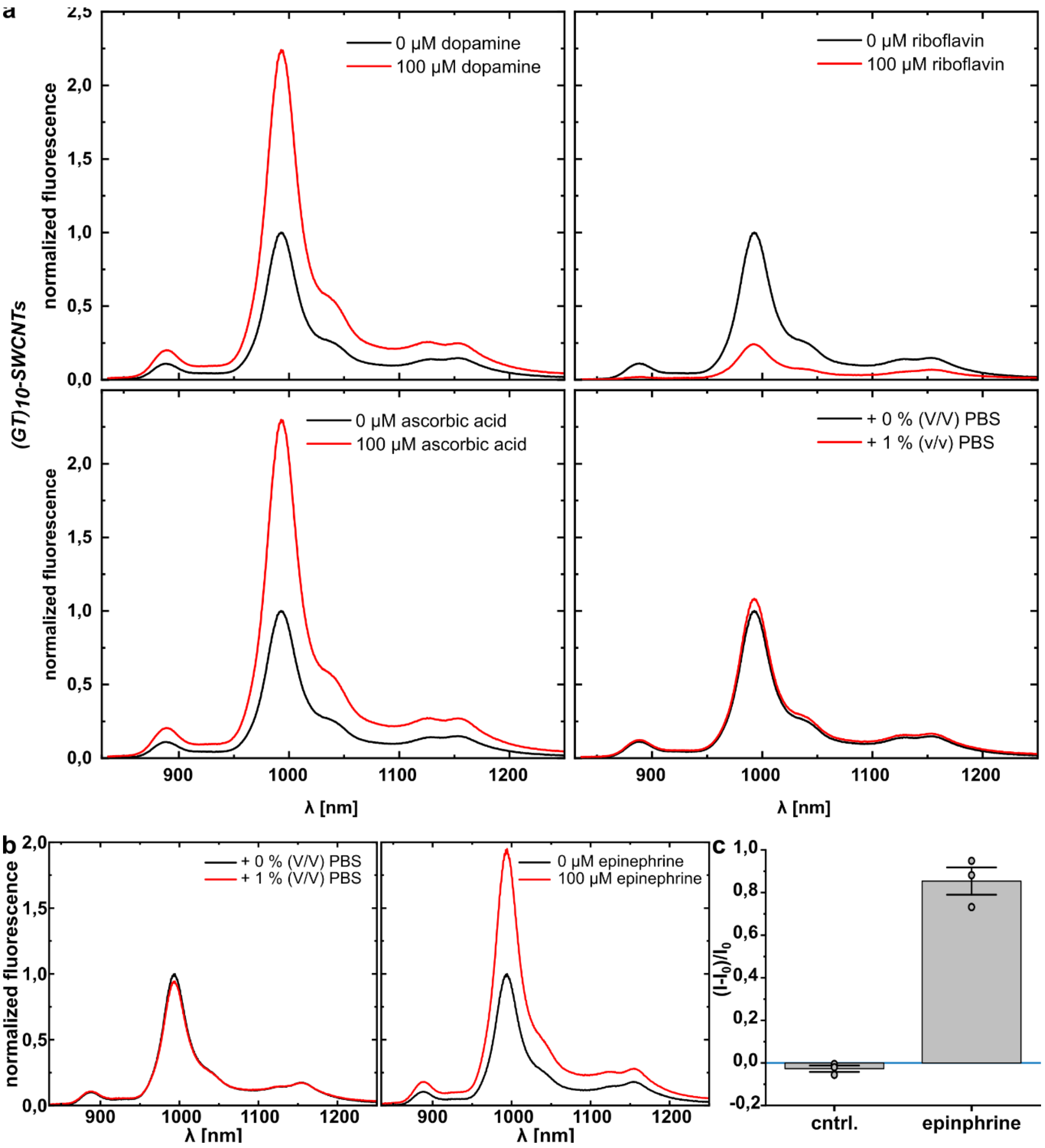

Supplementary Fig. 2. Mean (n = 3) fluorescence spectra of $(GT)_{10}$-SWCNT (single wall carbon nanotube) sensors before and after the addition of analytes in buffer (phosphate buffered saline, PBS). (a) Addition of dopamine, riboflavin, ascorbic acid, and buffer (PBS, control). (b) Fluorescence spectra before and after the addition of 1 % (V/V) PBS buffer as control and 100 µM epinephrine (end concentration). (c) Relative fluorescence change. Data are represented as mean values ± SEM (n=3).

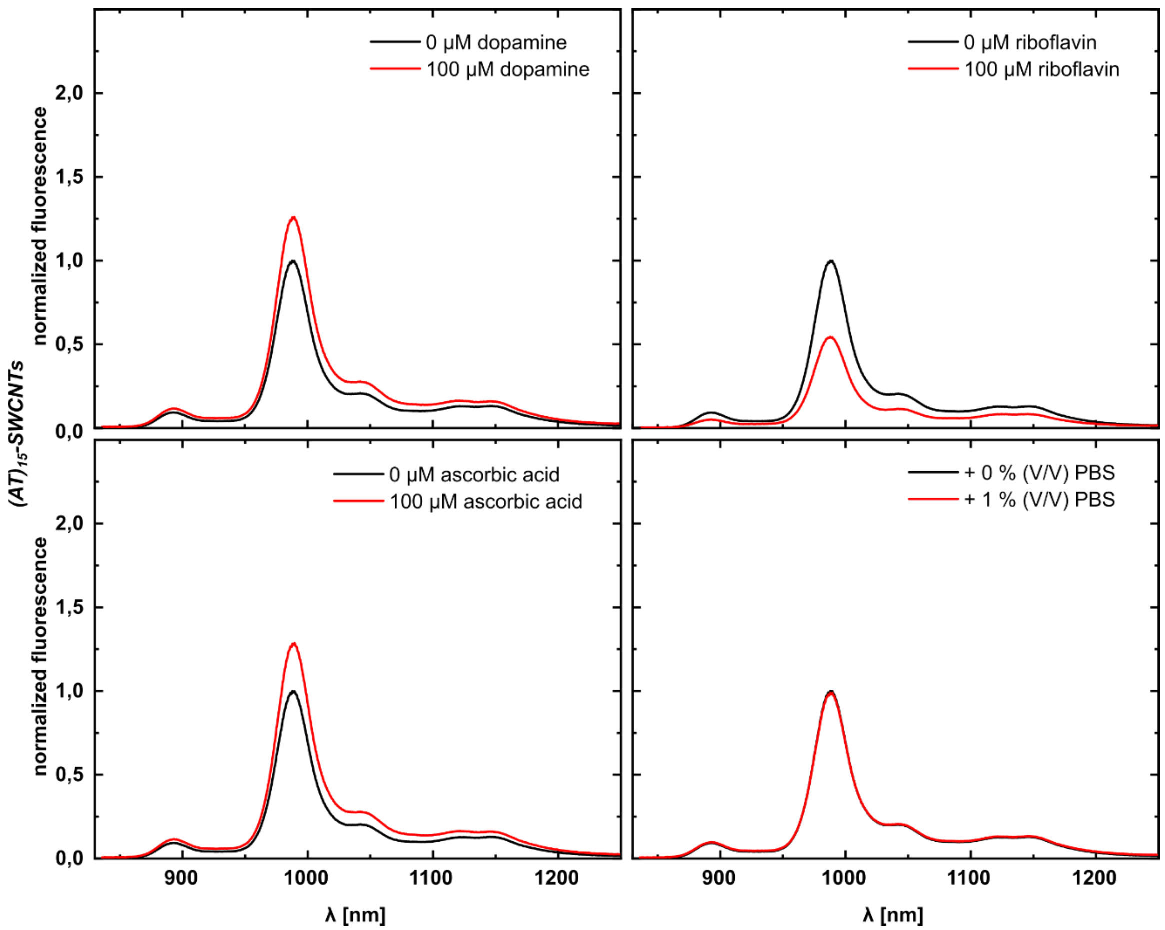

Supplementary Fig. 3. Mean (n = 3) fluorescence spectra of $(AT)_{15}$-SWCNT sensors before and after the addition of analytes in buffer (PBS).

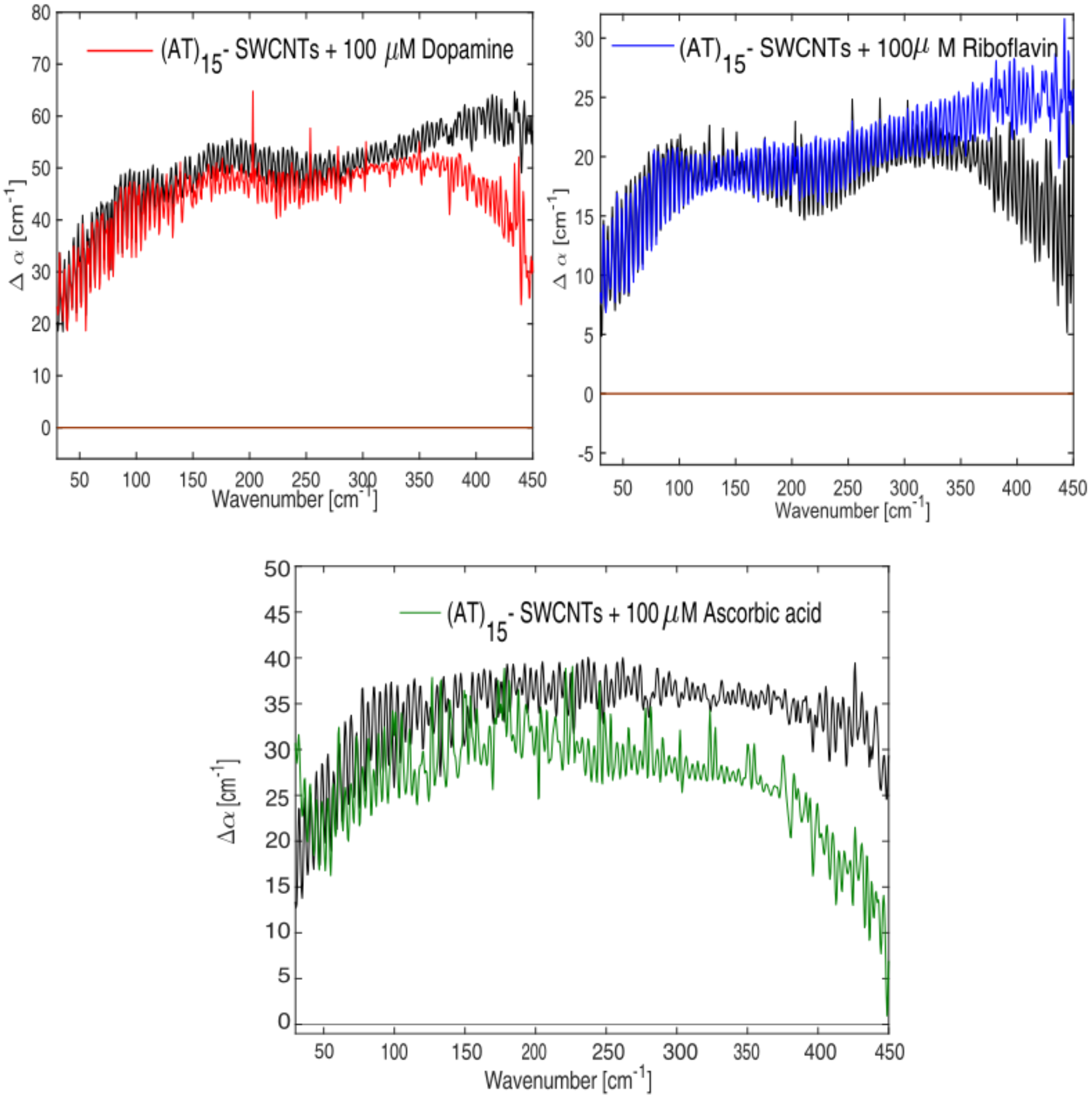

Supplementary Fig. 4. Plot of difference spectra, $\Delta\boldsymbol{\alpha}$ (= $\boldsymbol{\alpha}_{sample} - \boldsymbol{\alpha}_{water}$) vs wavenumber of DNA-SWCNTs (black) and of them with analytes (color coded). The DNA sequence used here is $(AT)_{15}$. The above spectra are not corrected for concentration. They are an average of multiple trials of distinct samples carried out on two days for each analyte. Concentration corrected data set available at the repository.

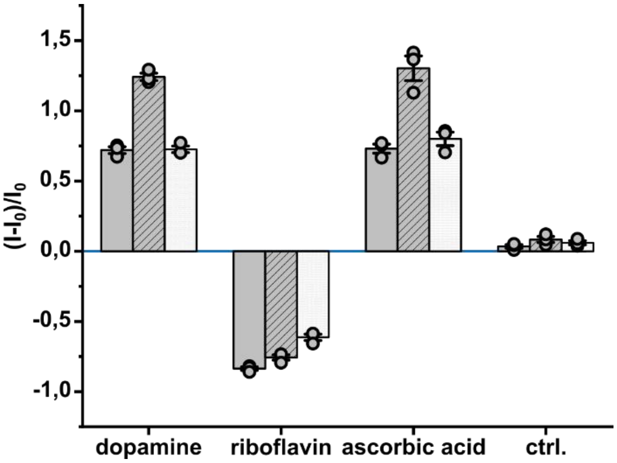

Supplementary Fig. 5. Relative change of fluorescence intensity of distinct SWCNT chirality upon the addition of 100 µM of the corresponding analyte. The chiralities are identified from the fluorescence spectrum of a $(GT)_{10}$-CoMoCAT SWCNT sample by peak position. Data are represented as mean values ± SEM (n=3).

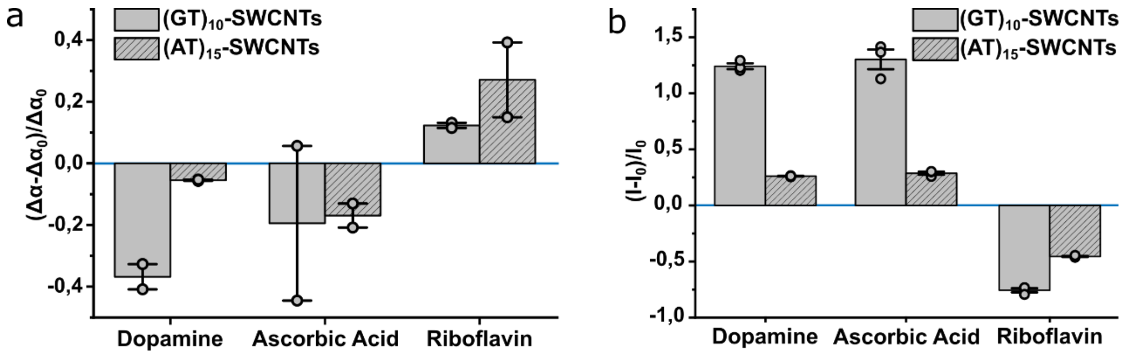

Supplementary Fig. 6. (a) Change in THz difference spectra upon addition of the analyte at ν = 150 cm^{-1} compared to the absorption of the solvated $(GT)_{10}$- and $(AT)_{15}$-SWCNTs. Data are represented as mean values ± SEM (n=3). (b) Fluorescence changes of $(GT)_{10}$ and $(AT)_{15}$-SWCNTs after the addition of 100 µM respective analyte. Data are represented as mean values ± SEM (n=3).

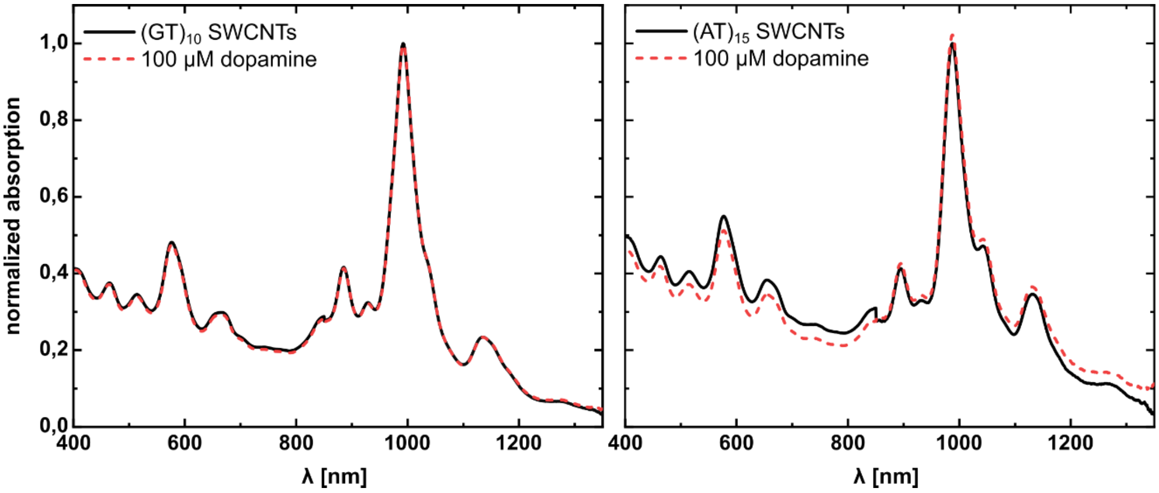

Supplementary Fig. 7. Representative normalized VIS and NIR absorption spectra of $(GT)_{10}$- and $(AT)_{15}$-SWCNT sensors before and after the addition of the neurotransmitter dopamine in buffer (PBS).

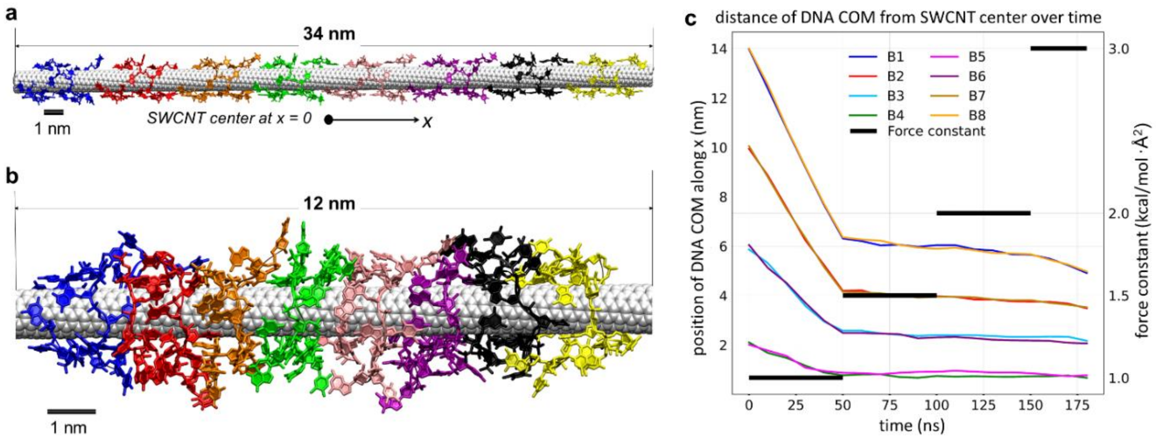

Supplementary Fig. 8. Initial confirmation of ssDNA-SWCNT conjugates. (a) eight $(GT)_{10}$ ssDNA strands were positioned initially to wrap a (6,5) SWCNT with a length of 340 Å. (b) Eight ssDNA mass centers were moved to designated places using the colvars method to occupy a length of 12 nm of the SWNT in total. (c) Distance of DNA molecule center of mass from the SWCNT center over time, as DNA molecules are forced to adsorb to the central part of the SWNT, 12 nm in length. The force constant used for moving DNA molecules towards the central part of the SWNT was increased gradually every 50 ns (black lines with values defined on the right vertical axis).

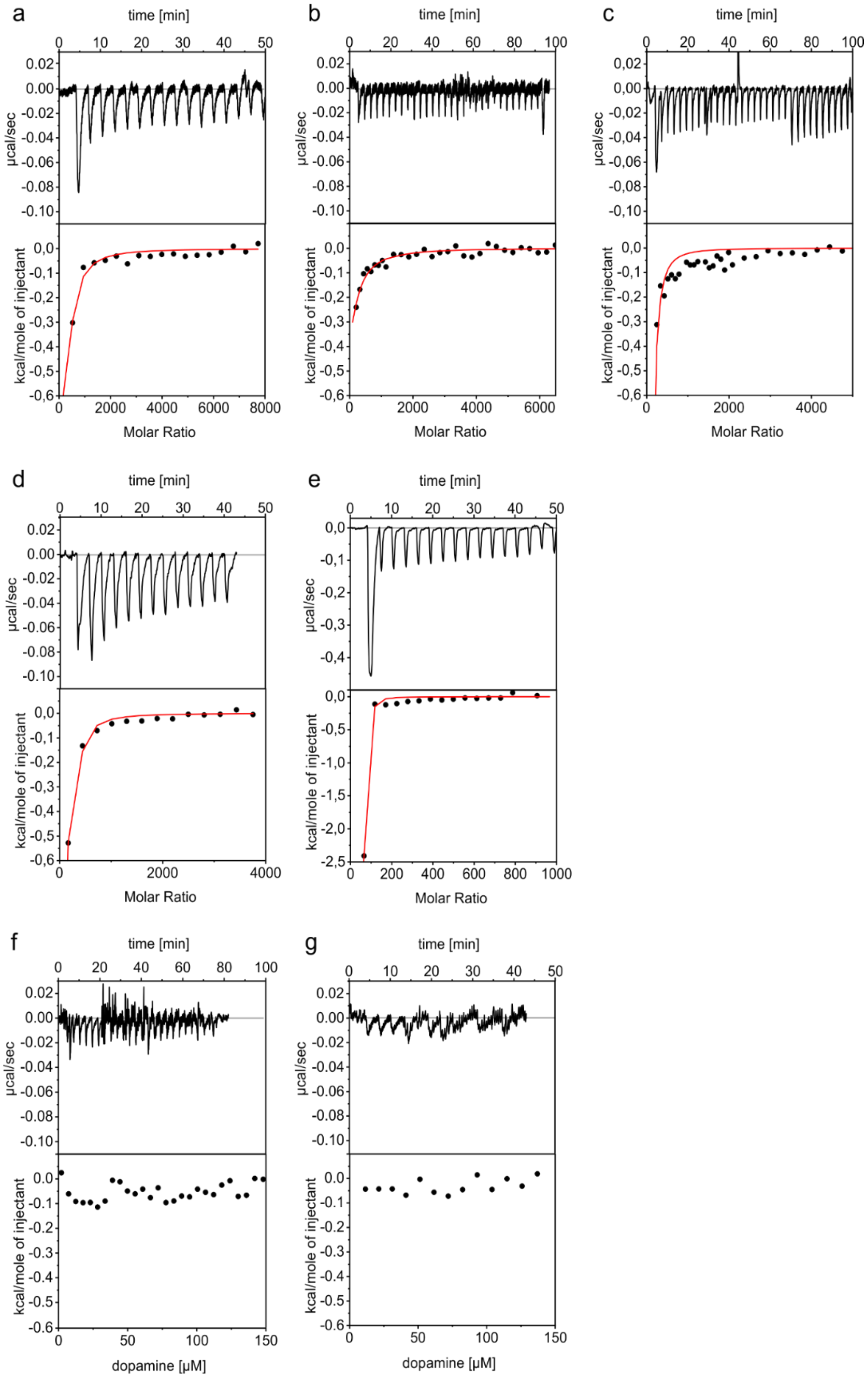

Supplementary Fig. 9. Isothermal Calorimetry Titration (ITC) of $(GT)_{10}$-SWCNTs with dopamine. Upper graph: c_{SWCNT} = 48 nM (a), 39 nM (b), 25 nM (c), 199 nM (d), 28,5 nM (e), $(GT)_{10}$-SWCNTs (f) and 1xPBS (g) were titrated with dopamine to a final concentration of 100 µM. Lower graph: Binding isotherms for the respective titrations plotted against the molar ratio dopamine to $(GT)_{10}$-SWCNTs. Binding isotherms were fitted with a one-site binding model (red).

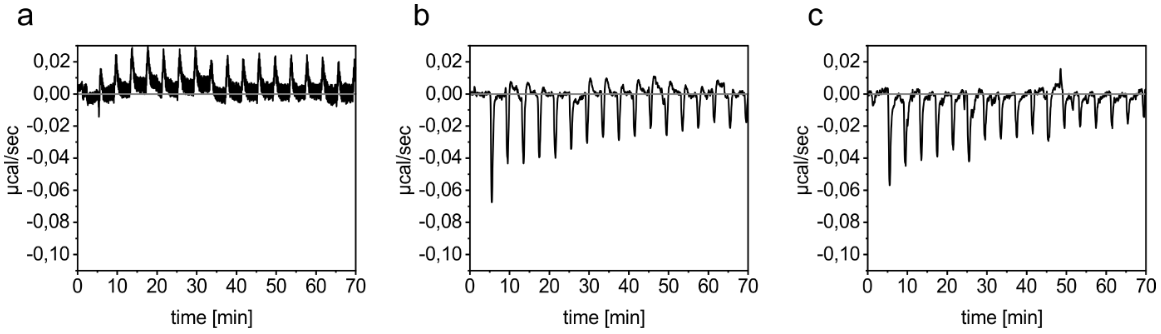

Supplementary Fig. 10. Isothermal Titration Calorimetry (ITC) of $(GT)_{10}$-SWCNTs with riboflavin. The raw data of the heating power is plotted. a. Control experiments were carried out with no $(GT)_{10}$-SWCNTs in the cell but only 1xPBS. (b + c) 300 µM solution of riboflavin was titrated to a 60 nM solution of $(GT)_{10}$-SWCNTs in the cell.

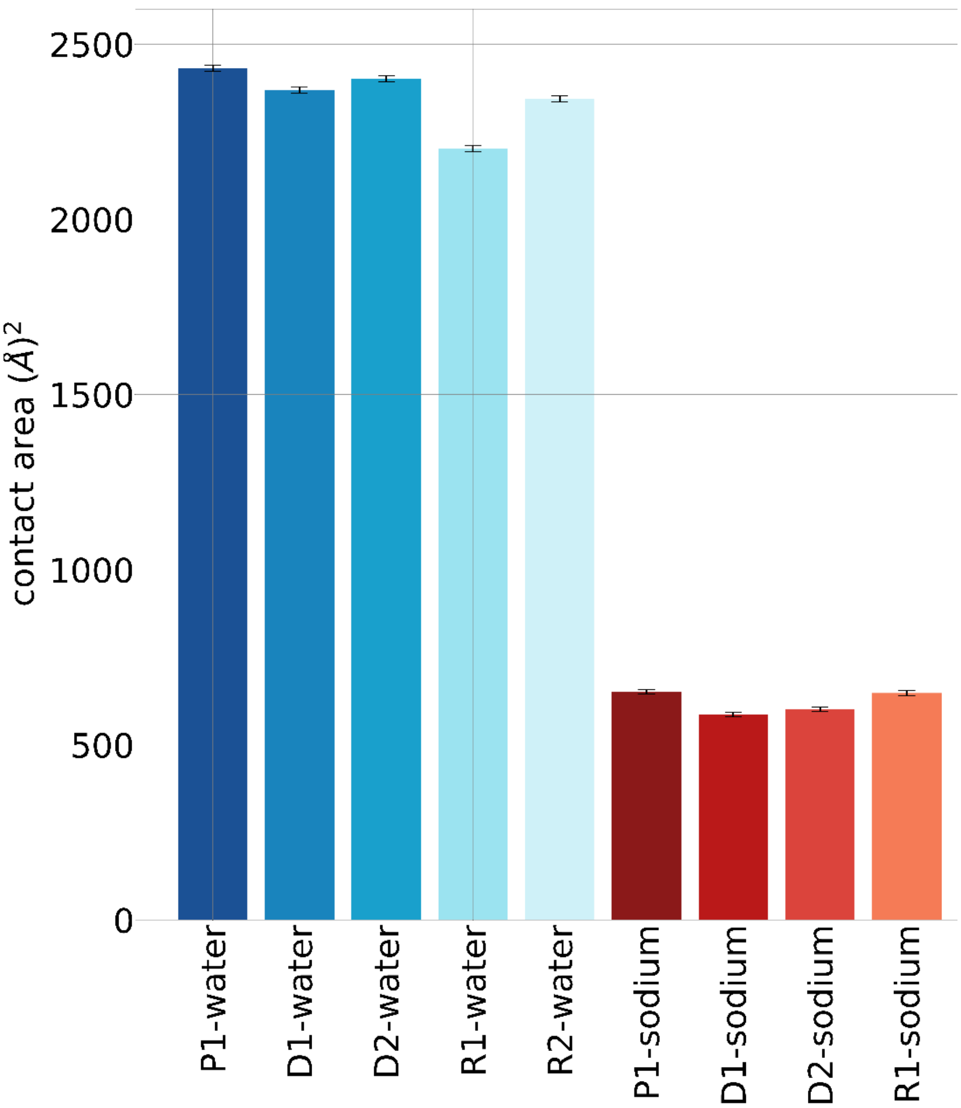

Supplementary Fig. 11. Bulk contact areas between the SWCNT surface and water or sodium ions. Data are presented as mean values ± SEM, with averaging performed over simulation times (3 μs).

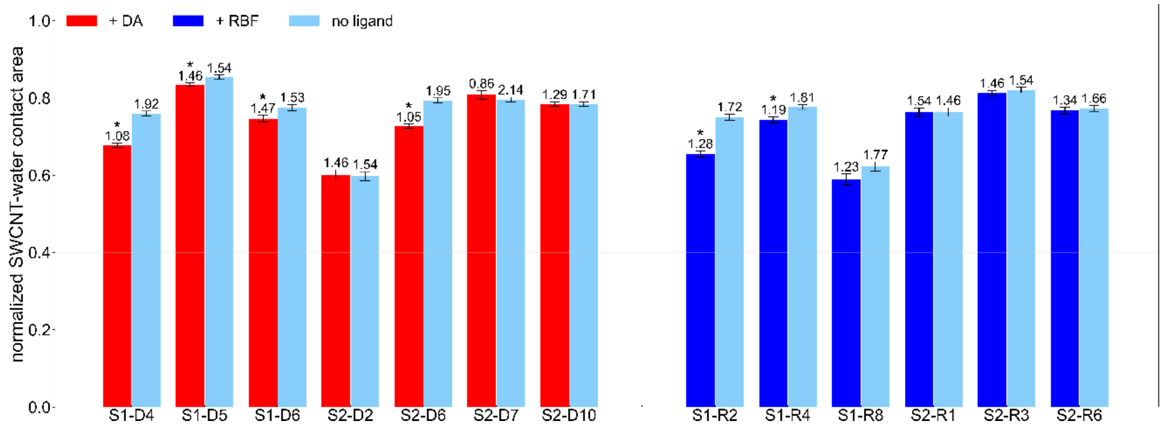

Supplementary Fig. 12. Analysis of local contact areas between the SWCNT surface segment and surrounding water molecules. Red and dark blue signify contact areas between the SWCNT segment and local water molecules when dopamine and riboflavin bind to the SWCNT, respectively. The asterisk (*) symbol designates ligands that adopt the first binding mode, which involves binding to SWCNT. Conversely, if the asterisk symbol is not present, it indicates that the ligand utilizes the second binding mode, binding to the DNA corona. Light blue bars represent contact areas between the SWCNT segment and local water molecules without ligand binding. Data are presented as mean values ± standard error, with averaging performed over simulation times indicated above the bars, where the times are reported in units of microseconds. The x-axis labels display the simulation system number and ligand ID, with contact areas normalized by considering the surface areas of SWCNT segments in the analyses.

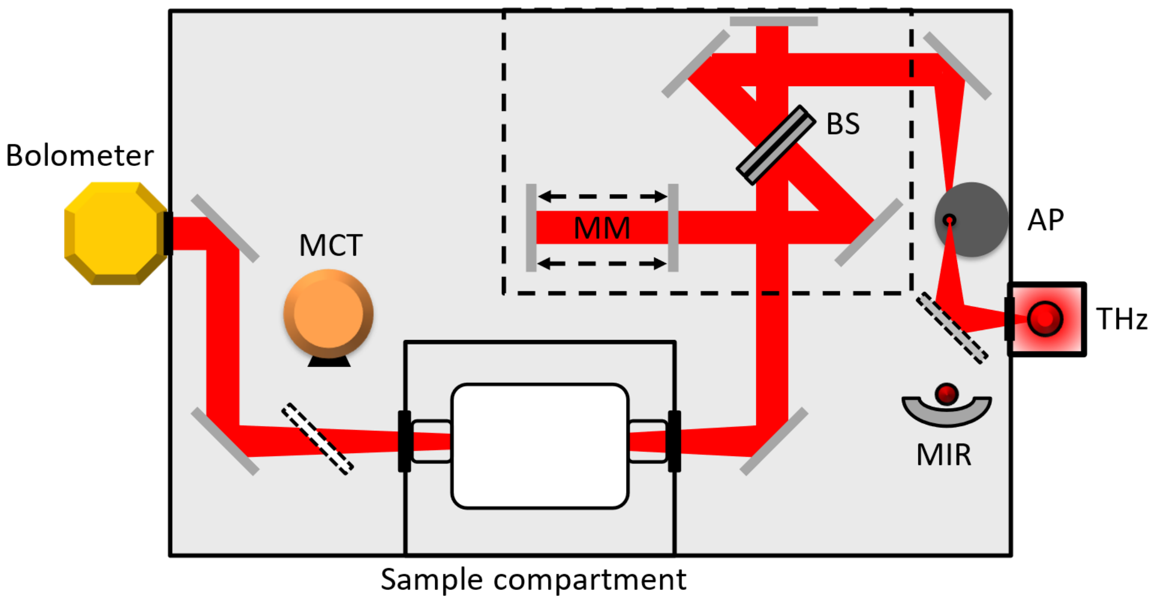

Supplementary Fig.13. Schematic of FTIR[10]. The sample compartment is temperature stabilized and purged with dry nitrogen to ensure stable operating conditions during measurements.

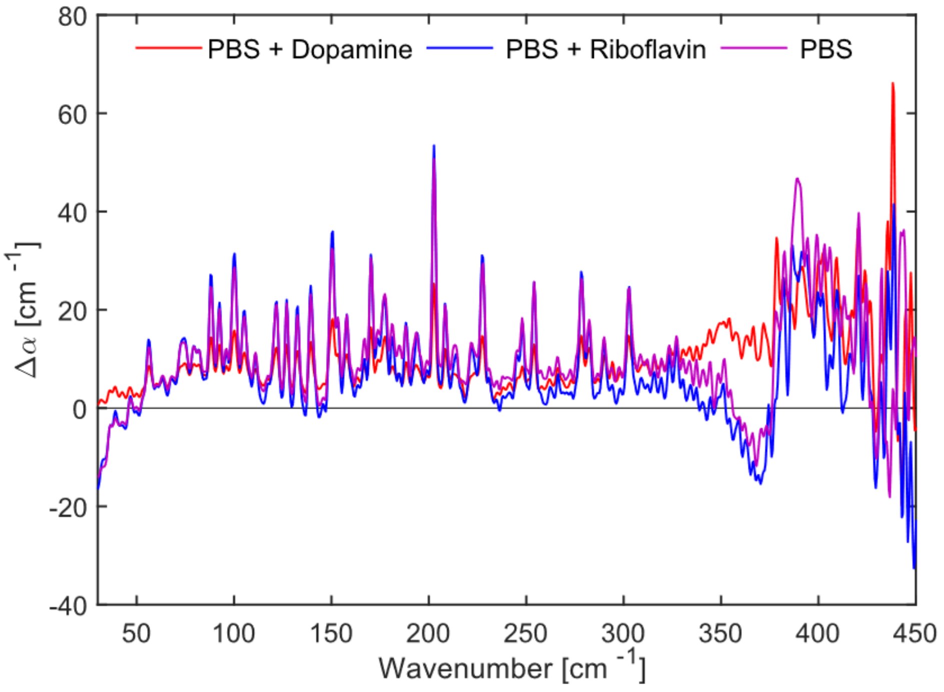

Supplementary Fig. 14. Plot of difference spectra, $\Delta\boldsymbol{\alpha}$ (= $\boldsymbol{\alpha}_{sample} - \boldsymbol{\alpha}_{water}$) vs wavenumber of PBS buffer (used for the preparation of the DNA-SWCNT samples) with and without analyte. Average of two measurements of distinct samples carried out on the same day.

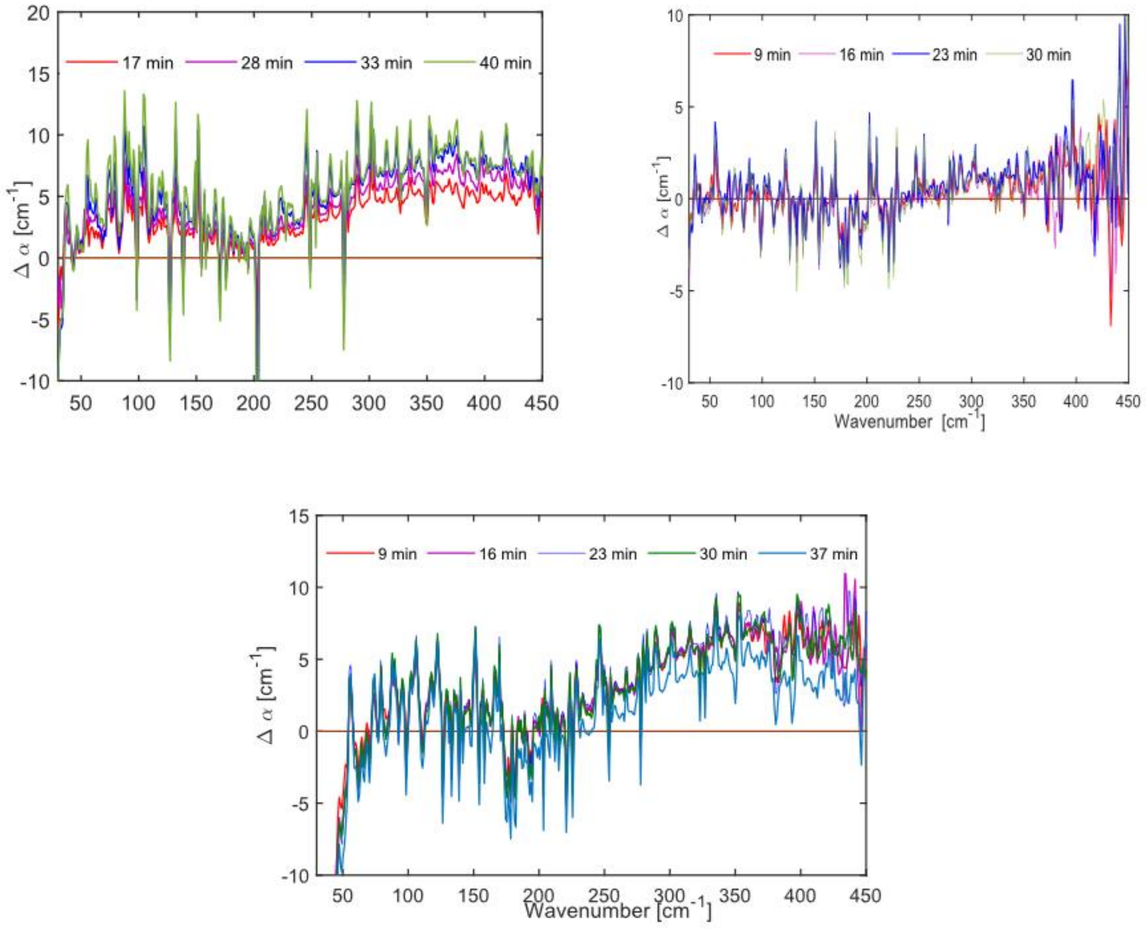

Supplementary Fig. 15. Plot of difference spectra, $\Delta\boldsymbol{\alpha}$ (= $\boldsymbol{\alpha}_n - \boldsymbol{\alpha}_1$) vs wavenumber of dopamine, riboflavin, and ascorbic acid respectively (left to right and below, all have a concentration of 100 µM). The spectrum measured at n^{th} time was subtracted from the first spectrum taken after the addition of analyte into the sample cell. The above spectra of each analyte were measured for a given amount of time with a time interval. We do not observe any shift in water around the analytes with time.

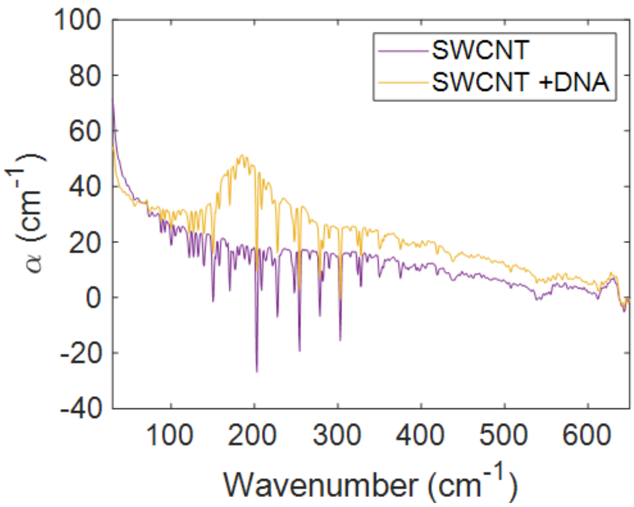

Supplementary Fig. 16. Plot of THz absorption spectra, $\boldsymbol{\alpha}$ of SWCNT (solid) and dried DNA-SWCNTs using ATR unit in the FTIR-THz setup. The spectra shown here are an average of two trials (for distinct samples) carried out on the same day.

Supplementary Table 1. The simulation results highlight only those ligands that exhibit both binding modes.

Simulation System No.	Ligand name	Binding modes	Number of carbons atoms in the gray region	ligand ID
1-DA	Dopamine	1	74	4
		1	112	5
		1	107	6
2-DA		2	31	2
		1	93	6
		2	40	7
		2	111	10
1-RBF	Riboflavin	1	45	2
		1	83	4
		2	23	8
2-RBF		2	45	1
		2	50	3
		2	19	6

Supplementary Table 2. Summary of the simulated DNA-SWCNT systems. Composition of DNA-SWCNT systems.

System name	Number of ligands	Ligand type	Number of water molecules	Number of Cl^- ions	Size of water box ($Å^3$)
pure-1	0	----	21337	58	120x80x80
dop-1	10	Dopamine	21180	68	
dop-2			21327	58	
ribo-1		Riboflavin	21337	58	
ribo-2			21337	58	

Supplementary Table 3. Key data and results of ITC experiments on titration of $(GT)_{10}$-SWCNTs with 100 µM dopamine. Outliers are marked with brackets.

c(SWCNTs) [nM]	Binding sites N	K_D [µM]
48	30,3	5,5
28,8	(173)	12
199	42,6	(0,3)
39	29,6	(0,2)
25	39	10
28,45	42	16,8
**MW±SD (w/o outlier)**	**36,7 ± 5,6**	**11,1 ± 4,1µM**

SI References

1. R. Nißler, *et al.*, Quantification of the Number of Adsorbed DNA Molecules on Single-Walled Carbon Nanotubes. *The Journal of Physical Chemistry C* **123**, 4837–4847 (2019).
2. F. Mann, N. Herrmann, D. Meyer, S. Kruss, Tuning Selectivity of Fluorescent Carbon Nanotube-Based Neurotransmitter Sensors. *Sensors* **17**, 1521 (2017).
3. T. Wiseman, S. Williston, J. F. Brandts, L.-N. Lin, Rapid measurement of binding constants and heats of binding using a new titration calorimeter. *Anal Biochem* **179**, 131–137 (1989).
4. A. G. Beyene, *et al.*, Ultralarge Modulation of Fluorescence by Neuromodulators in Carbon Nanotubes Functionalized with Self-Assembled Oligonucleotide Rings. *Nano Lett* **18**, 6995–7003 (2018).
5. J. C. Phillips, *et al.*, Scalable molecular dynamics on CPU and GPU architectures with NAMD. *J Chem Phys* **153**, 044130 (2020).
6. T. Darden, D. York, L. Pedersen, Particle mesh Ewald: An $N \cdot \log(N)$ method for Ewald sums in large systems. *J Chem Phys* **98**, 10089–10092 (1993).
7. W. Humphrey, A. Dalke, K. Schulten, VMD: Visual molecular dynamics. *J Mol Graph* **14**, 33–38 (1996).
8. J. B. Klauda, *et al.*, Update of the CHARMM All-Atom Additive Force Field for Lipids: Validation on Six Lipid Types. *J Phys Chem B* **114**, 7830–7843 (2010).
9. R. B. Best, *et al.*, Optimization of the Additive CHARMM All-Atom Protein Force Field Targeting Improved Sampling of the Backbone ϕ, ψ and Side-Chain χ_1 and χ_2 Dihedral Angles. *J Chem Theory Comput* **8**, 3257–3273 (2012).
10. K, Benedikt, May (2023), Local Hydration Upon Interaction of Complex Biomolecules by THz-ATR spectroscopy. Thesis (PhD). Ruhr University Bochum.
